# Using latent class analysis to develop a model of the relationship between socioeconomic position and ethnicity: cross-sectional analyses from a multi-ethnic birth cohort study

**DOI:** 10.1186/1471-2458-14-835

**Published:** 2014-08-12

**Authors:** Lesley Fairley, Baltica Cabieses, Neil Small, Emily S Petherick, Debbie A Lawlor, Kate E Pickett, John Wright

**Affiliations:** Bradford Institute for Health Research, Bradford Teaching Hospitals NHS Foundation Trust, Bradford, UK; Faculty of Medicine, Universidad del Desarrollo, Santiago, Chile; School of Health Studies, University of Bradford, Bradford, UK; MRC Centre for Causal Analyses in Translational Epidemiology, School of Social and Community Medicine, University of Bristol, Bristol, UK; Department of Health Sciences, University of York, York, UK

**Keywords:** Socioeconomic position, Ethnicity, Latent class analysis, Born in Bradford

## Abstract

**Background:**

Almost all studies in health research control or investigate socioeconomic position (SEP) as exposure or confounder. Different measures of SEP capture different aspects of the underlying construct, so efficient methodologies to combine them are needed. SEP and ethnicity are strongly associated, however not all measures of SEP may be appropriate for all ethnic groups.

**Methods:**

We used latent class analysis (LCA) to define subgroups of women with similar SEP profiles using 19 measures of SEP. Data from 11,326 women were used, from eight different ethnic groups but with the majority from White British (40%) or Pakistani (45%) backgrounds, who were recruited during pregnancy to the Born in Bradford birth cohort study.

**Results:**

Five distinct SEP subclasses were identified in the LCA: (i) "Least socioeconomically deprived and most educated" (20%); (ii) "Employed and not materially deprived" (19%); (iii) "Employed and no access to money" (16%); (iv) "Benefits and not materially deprived" (29%) and (v) "Most economically deprived" (16%). Based on the magnitude of the point estimates, the strongest associations were that compared to White British women, Pakistani and Bangladeshi women were more likely to belong to groups: (iv) "benefits and not materially deprived" (relative risk ratio (95% CI): 5.24 (4.44, 6.19) and 3.44 (2.37, 5.00), respectively) or (v) most deprived group (2.36 (1.96, 2.84) and 3.35 (2.21, 5.06) respectively) compared to the least deprived class. White Other women were more than twice as likely to be in the (iv) "benefits and not materially deprived group" compared to White British women and all ethnic groups, other than the Mixed group, were less likely to be in the (iii) "employed and not materially deprived" group than White British women.

**Conclusions:**

LCA allows different aspects of an individual’s SEP to be considered in one multidimensional indicator, which can then be integrated in epidemiological analyses. Ethnicity is strongly associated with these identified subgroups. Findings from this study suggest a careful use of SEP measures in health research, especially when looking at different ethnic groups. Further replication of these findings is needed in other populations.

## Background

Socioeconomic position (SEP), is a multidimensional concept that includes both resource based components (such as income and material wealth) and prestige based components (e.g. education, occupation, societal position amongst peers) and, ideally, takes account of these across the life course [1–4]. It has been emphasised that, whether SEP is being used as the main exposure variable in epidemiological research or as a potential confounder, combining several of these different components is important to fully capture SEP variation [1–3]. However while the definition clearly indicates the need to include multiple components in its assessment, single measures of SEP (such as occupation or educational attainment) are frequently used in research. In part this may reflect the availability of only one or two measurements in a given study, but it may also reflect lack of certainty about how to combine a number of different measurements appropriately, particularly where there are complex patterns of missing data between measurements.

There is also uncertainty about how to determine which measurements best capture the full concept of SEP for different groups, based for example on ethnicity, gender or age [1–6]. For example, women who do not work outside of the home may be classified according to their husband’s occupation, but this may or may not reflect their personal material wealth or social standing; similarly previous occupation may not be a good measure of SEP for those past retirement age. Educational attainment might have markedly different meaning for people from different ethnic groups (particularly if education has been received in different countries) [4, 5]. These differences may also be reflected in patterns of missing data, for example there may be gender and ethnic differences in ability or willingness to answer questions about total household income.

Given its relevance to health research it is important to explore how to efficiently combine multiple measures of SEP together in a meaningful way to the population being researched. Latent class analysis (LCA), is an established statistical method that allows the classification of individuals into groups based on conditional probabilities; within each group individuals will have a similar pattern of response to categorical variables [7]. This approach can also incorporate responses that other analyses may treat as missing data by using a full information maximum likelihood (FIML) approach. This approach does not impute missing values but uses all the available information for each individual to provide maximum likelihood estimations [8, 9]. This method produces unbiased parameter estimates and standard errors under missing at random and missing completely at random assumptions. LCA is also flexible in the number and types of variables that can be included.

LCA is a data driven approach and is increasingly being applied to health related data. LCA has been used to identify subgroups of study populations based on SEP in different studies in the UK [10, 11] the USA [12, 13] and the Philippines [14]. These studies found that between 3 and 7 classes best described the SEP profiles of study participants. To our knowledge no previous study has used LCA to identify subgroups of the population in relation to SEP and explore how these classes relate to, or are influenced by, ethnicity.

In order to examine ethnic differences in health it is important to make appropriate adjustment for SEP, assuming that once SEP is considered any ethnic group differences are due to factors linked to ethnicity such as cultural and genetic differences. It is unclear whether gradients in health by SEP in particular ethnic groups are not always found due to poor and inappropriate measurements of SEP for such groups. The aim of this paper was to describe how latent class analysis can be used to help us understand and combine several measures of SEP into one measure in a multi-ethnic population. We did this in two ways. First, we identified different SEP subgroups of a population of women who were recruited during pregnancy from a largely deprived and ethnically diverse city in the North of England and examined the association of ethnicity to these subgroups. This allowed all of the eight different ethnic groups recruited to the cohort to be included in the analyses and examined whether women from different ethnic groups were more or less likely to belong to different SEP subgroups that have been robustly defined using a wide-range of SEP components. Second, we used the LCA approach to identify subgroups based on SEP separately within the two largest ethnic groups in the cohort; White British (comprising 40% of the participants) and Pakistani origin women (45%). This allowed us to examine whether components of SEP would aggregate differently in two distinct ethnic groups. We were only able to do this for the two largest groups as the other six ethnic groups contained too few participants for robust analyses.

## Methods

### Data

The Born in Bradford (BiB) study is a longitudinal multi-ethnic birth cohort study aiming to examine the impact of environmental, psychological and genetic factors on maternal and child health and wellbeing [15]. Bradford is a city in the North of England with high levels of deprivation and ethnic diversity. Pregnant women were recruited at 26-28 weeks gestation. For those consenting, a baseline questionnaire was completed via an interview with a trained study administrator. The full BiB cohort recruited 12453 mothers who had 13776 pregnancies between 2007 and 2010, of whom 11396 (82.7%) completed the baseline questionnaire. The cohort is broadly characteristic of the city’s maternal population [15]. Ethical approval for the data collection was granted by Bradford Research Ethics Committee (Ref 07/H1302/112).

### Measures of socioeconomic position

Within the BiB cohort 19 components of SEP were collected by interview at recruitment. These are described in Table 1 and in the text below.

**Table 1:** **Summary of SEP variables used in latent class analysis**

Variable description	Category level in analysis
Woman’s employment status	Currently employed, previously employed, never employed,
Baby’s father’s employment status	Non- manual, manual, self-employed, student, unemployed, don’t know
Mother’s education	<5 GCSE equivalent, 5 GCSE equivalent, A-level equivalent, higher than A-level, other, don’t know, foreign unknown
Baby’s father’s education	<5 GCSE equivalent, 5 GCSE equivalent, A-level equivalent, higher than A-level, other, don’t know, foreign unknown
Subjective poverty	No, yes
Being in receipt of means tested benefits	No, yes
Up to date with bills	Yes, no, don’t know
Housing tenure	Owns outright, mortgage, lives rent free, private landlord, social housing, other, don’t know
Able to afford a holiday from home for at least one week once a year	Have or don’t want or need, can’t afford
Able to afford family and friends for a drink or meal at least once a month	Have or don’t want or need, can’t afford
Able to afford two pairs of all weather shoes	Have or don’t want or need, can’t afford
Able to afford enough money to keep home in decent state of decoration	Have or don’t want or need, can’t afford
Able to afford household contents insurance	Have or don’t want or need, can’t afford
Able to afford money to make regular savings of £10 a month	Have or don’t want or need, can’t afford
Able to afford money to replace any worn out furniture	Have or don’t want or need, can’t afford
Able to afford money to replace or repair major electrical goods	Have or don’t want or need, can’t afford
Able to afford a small amount of money to spend on yourself each week	Have or don’t want or need, can’t afford
Able to afford a hobby or leisure activity	Have or don’t want or need, can’t afford
In winter are you able to keep home warm enough	Have or don’t want or need, can’t afford

Over a quarter (27%) of all women reported they had never been employed, therefore we were unable to assign an occupational social class to these women. We did, however, include women’s employment as a categorical variable: currently employed, previously employed and never employed.

The baby’s father’s occupation was coded based on the National Statistics Socio-Economic Classification (NS-SEC) classification [16]. Categories were then collapsed into the following: non-manual employment (including modern professional occupations, clerical and intermediate occupations, senior managers or administrators, middle or junior managers, traditional professional occupations and technical and craft occupations), manual employment (including semi-routine manual and service occupations and routine manual and service occupations), self-employed, student, unemployed (including long term sick) and don’t know. We could not use the exact NS-SEC classification, as for some fathers we only had information to indicate that they were self-employed and did not have further information about the type of work they did.

The highest educational qualification obtained by the woman and the baby’s father was recorded along with the country it was obtained in. In England pupils sit General Certificate of Secondary Education (GCSE) examinations in different subjects usually at age 14-16, receiving 5 or more GCSEs is usually a requirement for undertaking Advanced level (A-level) studies, which are examinations in different subjects usually taken at age 16-18 before attending university. We equivalised the highest educational qualifications (based on the qualification received and the country obtained) into one of seven categories using UK National Academic Recognition Information Center [17] : <5 GCSE equivalent, 5 GCSE equivalent, A- level equivalent, higher than A-level equivalent, other, foreign unknown and don’t know. The foreign unknown category relates to responses given that referred to qualifications obtained overseas with insufficient information provided to determine their equivalence and the don’t know response relates to the mother or father responding "don’t know" during interview.

We included the responses to three questions about the women’s financial situation. Women were asked how difficult they were finding it to get by financially and were classified as subjectively poor if they responded "finding it very difficult" or "finding it quite difficult". Being in receipt of means tested benefits was defined as receiving any of the following: income support, income tested jobs seekers allowance, working families tax credit or housing benefit [18]. Finally we included a categorical variable to indicate if the women were up to date with all their household bills.

Eleven questions about ownership of material items and goods based on questions from the Households Below Average Income Survey [19] were asked. The questions asked if they were able to have; a holiday from home for at least one week once a year (not including staying with relatives in their home), friends and family round for a drink or meal at your house at least once a month, two pairs of all weather shoes, enough money to keep your home in a decent state of repair, household contents insurance, money to make regular savings of £10 a month, money to replace any worn out furniture, money to replace or repair major electrical goods, a small amount of money to spend each week on yourself, a hobby or leisure activity, in winter are you able to keep your home warm enough. All the responses to these questions were coded as either "have or don’t want or need" or "can’t afford".

Housing tenure was categorised into one of seven groups; owns outright, owns with a mortgage, lives rent free, owned by a private landlord, living in social housing, other and don’t know.

### Ethnicity

Questions relating to ethnicity were based on guidance from the Office for National Statistics [20], and comprised one question asking which ethnic group the mother considered themselves as belonging to (White, Mixed, Black or Black British, Asian or Asian British, Chinese or Other), followed by a further question, based on their response, about cultural background. This resulted in 8 ethnic groups in this analysis; White British, White Other, Mixed, Black, Indian, Pakistani, Bangladeshi and Other.

### Other characteristics

Although few characteristics could influence a woman’s ethnicity and therefore confound the association between ethnicity and SEP we felt it was important to consider two key characteristics that may be relevant in explaining the association between ethnicity and SEP. Several of the SEP indicators may be dependent on age and marital status and the distributions of these two variables differed by ethnicity, therefore we felt it was important to explore the effect of adjusting for these in our models. Woman’s age at recruitment into the study was categorised as <21 years, 21-34 years or 35+ years. Marital and cohabitation status were coded as married, cohabiting or not cohabiting.

### Missing data

Women with missing data on at least one of the covariables (ethnicity, age and marital and cohabitation status) were excluded from all analysis, 49 women had missing ethnicity data and 25 had missing data on marital and cohabitation status. In total 70 cases (0.6%) were excluded from analysis, leaving a total of 11326 women in the study sample.

## Statistical analysis

### Latent Class Analysis

Latent class analysis (LCA) was used to define groups of women with similar SEP profiles across the 19 determinants of SEP. LCA is a statistical method that allows the classification of individuals into groups based on conditional probabilities, within each class individuals will have a similar pattern of response to categorical variables [7]. This approach can also incorporate responses that other analyses may treat as missing data using the FIML approach [8, 9]. The main assumption for LCA is conditional independence, that is within each class all measures are independent as all correlation between the variables is explained through the class structure.

Latent class models with 2 to 10 classes were fitted to items measuring different aspects of the women’s SEP. Variables were entered into the models as either binary or nominal variables. Latent Class Analyses were carried out in Mplus V6 [21]. Criteria used to select the final LCA model [22] included the change in likelihood between models, Bayesian Information criterion (BIC) and entropy. The percentage change in the log-likelihood was compared for each model, selecting models where there was not too much discernable difference by adding another class. BIC is a measure of model fit with penalization for additional classes; models with lower values are considered a better model fit. Entropy measures how well an individual fits into a specific class with values ranging from 0 to 1 with values closer to 1 indicating better fit. We also considered the interpretability of the chosen model [23] by examining the probability of each level of the SEP indicators in each class to try to establish which variables distinguish each group from the others. After selecting the final LCA model, the posterior probability of belonging to each group can be obtained for each individual.

### Association of ethnicity with SEP latent class membership

Multinomial regression was used to assess the association between ethnicity and membership of the latent classes. The least socioeconomically deprived class was chosen as the reference category and models were weighted by probability of class membership. Unadjusted and adjusted models (adjusting for woman’s age and marital status) were fitted. Coefficients from these models were exponentiated to obtain relative risk ratios (RRR) with 95% confidence intervals. Multinomial regression was carried out in Stata 12 [24].

### Stratified LCA models for White British and Pakistani women only

As our sample was predominantly made up of two ethnic groups (40% White British and 45% Pakistani) we further explored the relationship between SEP indicators within these two groups only as the other six ethnic groups contained too few participants for robust analyses.

Latent class models were also run for the White British and Pakistani groups separately using the same methodology as described above.

## Results

### Study sample

Table 2 describes the study sample; 40% of the women were White British and 45% were of Pakistani origin, 7% of women were under 20 years of age and 66% were married and living with a partner.

**Table 2:** **Characteristics of study population**

Variable	Category	n	%
Woman’s ethnic group	White British	4480	39.6
	White Other	302	2.7
	Mixed	180	1.6
	Black	249	2.2
	Indian	438	3.9
	Pakistani	5117	45.2
	Bangladeshi	263	2.3
	Other	297	2.6
Woman’s age	<20 years	814	7.2
	20-34 years	9164	80.9
	35+ years	1348	11.9
Marital and cohabitation status	Married and living with partner	7451	65.8
	Not married and living with partner	2015	17.8
	Not living with partner	1860	16.4
Woman’s employment status	Currently employed	4987	44.0
	Previously employed	3231	28.5
	Never employed	3093	27.3
	Missing	15	0.1
Woman’s husband/partner employment status	Non- manual	4338	38.3
	Manual	3687	32.6
	Self-employed	1627	14.4
	Student	185	1.6
	Unemployed	857	7.6
	Don’t know	137	1.2
	Missing	495	4.4
Woman’s education	<5 GCSE equivalent	2438	21.5
	5 GCSE equivalent	3469	30.6
	A-level equivalent	1638	14.5
	Higher than A-level	2892	25.5
	Other	625	5.5
	Don’t know	127	1.1
	Foreign Unknown	112	1.0
	Missing	25	0.2
Baby’s father’s education	<5 GCSE equivalent	1731	15.3
	5 GCSE equivalent	2710	23.9
	A-level equivalent	1156	10.2
	Higher than A-level	2848	25.1
	Other	510	4.5
	Don’t know	2228	19.7
	Foreign Unknown	110	1.0
	Missing	33	0.3
Subjective poverty	No (not subjectively poor)	10395	91.8
	Yes (subjectively poor)	862	7.6
	Missing	69	0.6
Means tested benefits	No	6673	58.9
	Yes (receipt of means tested benefits)	4618	40.8
	Missing	35	0.3
Up to date with bills	Yes	9793	86.5
	No	1174	10.4
	Don’t know	317	2.8
	Missing	42	0.4
Housing tenure	Owns outright	1580	14.0
	Mortgage	5149	45.5
	Rent free	858	7.6
	Private landlord	2206	19.5
	Social housing	1238	10.9
	Other	144	1.3
	Don’t know	128	1.1
	Missing	23	0.2
Holiday from home for at least one week once a year	Have or don’t want or need	7144	63.1
	Can’t afford	4084	36.1
	Missing	98	0.9
Family and friends for a drink or meal at least once a month	Have or don’t want or need	11081	97.3
	Can’t afford	232	2.1
	Missing	76	0.7
Two pairs of all weather shoes	Have or don’t want or need	11035	97.4
	Can’t afford	244	2.2
	Missing	47	0.4
Enough money to keep home in decent state of decoration	Have or don’t want or need	10181	89.9
	Can’t afford	1053	9.3
	Missing	92	0.8
Household contents insurance	Have or don’t want or need	8715	77.0
	Can’t afford	1320	11.7
	Missing	1291	11.4
Money to make regular savings of £10 a month	Have or don’t want or need	8094	71.5
	Can’t afford	2395	21.2
	Missing	837	7.4
Money to replace any worn out furniture	Have or don’t want or need	8151	72.0
	Can’t afford	2992	26.4
	Missing	183	1.6
Money to replace or repair major electrical goods	Have or don’t want or need	8553	75.5
	Can’t afford	2595	22.9
	Missing	178	1.6
A small amount of money to spend on yourself each week	Have or don’t want or need	9406	83.1
	Can’t afford	1839	16.2
	Missing	81	0.7
A hobby or leisure activity	Have or don’t want or need	10580	93.4
	Can’t afford	664	5.9
	Missing	82	0.7
In winter are you able to keep home warm enough	Have or don’t want or need	10852	95.8
	Can’t afford	394	3.5
	Missing	80	0.7

## LCA results

The 3, 4 and 5 class models showed similar model fit in terms of likelihood, BIC and entropy (Table 3); however based on interpretability we decided that the 5 class model best described the different socioeconomic characteristics of the women in this study. We used the profiles of the different classes to describe the different groups and assign brief labels to them.

**Table 3:** **Model fit statistics for Latent Class analysis models with 1 to 10 classes**

Number of classes	Log-likelihood	% reduction in L	BIC	Entropy
1	-141158	0	282689	1.0
2	-129500	8.3	259757	0.86
3	-127143	9.9	255426	0.77
4	-126197	10.6	253915	0.76
5	-125536	11.1	252977	0.77
6	-125012	11.4	252312	0.74
7	-124654	11.7	251978	0.75
8	-124335	11.9	251722	0.75
9	-124055	12.1	251545	0.76
10	-123816	12.3	251450	0.75

**Figure 1:**
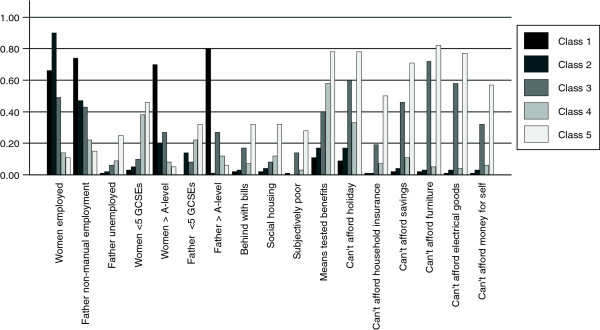
**Summary of selected predicted probabilities for levels of SEP indicators from 5 class model for all women.** Class 1: "Least socioeconomically deprived and most educated" (20%, n = 2231 (based on most likely class membership)). Class 2: "Employed, not materially deprived" (19%, n = 2248). Class 3: "Employed, no access to money" (16%, n = 1722). Class 4: "Benefits not materially deprived" (29%, n = 3325). Class 5: "Most economically deprived" (16%, n = 1800).

We assigned the following labels to the groups; (i) "Least socioeconomically deprived and most educated" (20% n = 2231 (based on most likely class membership)), (ii) "Employed and not materially deprived" (19%, n = 2248), (iii) "Employed and no access to money" (16%, n = 1722), (iv) "Benefits and not materially deprived" (29%, n = 3325) and (v) "Most economically deprived" (16%, n = 1800). The probabilities for selected categories of the SEP indicators within each class are shown in Figure 1 and a brief description of each group is given in Table 4.

**Table 4:** **Description of latent classes from 5 class model solution for all ethnic groups**

Class	Size of class (n†)	Description
**"Least socioeconomically deprived and most educated"**	20% (n = 2231)	Women currently and previously employed
		Father non-manual employment
		Women and fathers highly educated
		Up to date with bills
		Mortgage
		Not subjectively poor
		Not receiving means tested benefits
		Not materially deprived
**"Employed, not materially deprived"**	19% (n = 2248)	Women currently employed
		Father manual and non-manual employment
		Women and father medium levels of education
		Up to date with bills
		Mortgage
		Not subjectively poor
		Not receiving means tested benefits
		Not materially deprived
**"Employed, no access to money"**	16% (n = 1722)	Women currently and previously employed
		Father manual and non-manual employment
		Women and fathers medium levels of education
		Moderate behind with bills
		Mortgage and private renting
		Moderate subjective poverty
		Moderate receipt of means tested benefits
		Materially deprived in particular can’t afford holidays, money to replace goods and savings
**"Benefits and not materially deprived"**	29% (n = 3325)	Women low current employment,
		Father manual employment and self-employed
		Women and fathers low levels of education, fathers education high don’t know response
		Up to date with bills
		Owns house outright
		Not subjectively poor
		High receipt of means tested benefits
		Not materially deprived
**"Most economically deprived"**	16% (n = 1800)	Women low current employment
		Father manual employment and unemployed
		Women and fathers low levels of education, fathers education high don’t know response
		Behind with bills
		Private renting and social housing
		Subjectively poor
		Highest receipt of means tested benefits
		Materially deprived

† n based on most likely class membership

### Association between ethnicity and LCA subgroups

In the multinomial models the "least socioeconomically deprived and most educated" group was used as the reference category (Table 5). Compared to White British women, all ethnic groups other than the Mixed group were less likely to be in the "employed and not materially deprived" group. Women of Mixed, Pakistani and Bangladeshi ethnicities were more likely to be in the "benefits and not materially deprived group" compared to White British women (adjusted RRR = 2.37 (95% CI 1.31 to 4.27), RRR = 5.24 (95% CI 4.44 to 6.19) and RRR = 3.44 (95% CI 2.37 to 5.00) respectively). The risk of being in the most economically deprived group compared to the least deprived and most educated group was lower for women of White Other, Indian, Other ethnicities (adjusted RRR = 0.52 (95% CI 0.33 to 0.80), RRR = 0.26 (95% CI 0.15 to 0.45) and RRR = 0.57 (95% CI 0.38 to 0.85) respectively) compared to White British women. The risk of membership of this class was greater for women of Pakistani and Bangladeshi origin compared to White British women (adjusted RRR = 2.36 (95% CI 1.96 to 2.84), RRR = 3.35 (95% CI 2.21 to 5.06) respectively).

**Table 5:** **Relative Risk Ratios (RRR) and 95% CI results for membership of the latent classes compared to the "Least socioeconomically deprived and most educated" group from multinomial models weighted by probability of class membership**

	Employed not materially deprived				Employed no access to money				Benefits not materially deprived				Most deprived			
	Unadjusted		Adjusted†		Unadjusted		Adjusted †		Unadjusted		Adjusted †		Unadjusted		Adjusted †	
	RRR	95% CI	RRR	95% CI	RRR	95% CI	RRR	95% CI	RRR	95% CI	RRR	95% CI	RRR	95% CI	RRR	95% CI
**Ethnic group**																
White British	1		1		1		1		1		1		1		1	
White Other	0.50	(0.37,0.69)	0.58	(0.42,0.80)	0.81	(0.58,1.13)	1.04	(0.73,1.46)	0.39	(0.26,0.57)	0.54	(0.36,0.82)	0.31	(0.21,0.47)	0.52	(0.33,0.80)
Mixed	1.37	(0.77,2.45)	1.33	(0.74,2.39)	1.76	(0.94,3.28)	1.52	(0.80,2.89)	3.15	(1.80,5.51)	2.37	(1.31,4.27)	2.34	(1.32,4.15)	1.68	(0.91,3.10)
Black	0.19	(0.12,0.30)	0.23	(0.15,0.37)	0.96	(0.68,1.37)	1.22	(0.85,1.76)	0.33	(0.21,0.53)	0.47	(0.29,0.76)	0.75	(0.53,1.07)	1.03	(0.70,1.53)
Indian	0.22	(0.17,0.29)	0.35	(0.26,0.47)	0.33	(0.24,0.44)	0.66	(0.48,0.92)	0.36	(0.27,0.48)	0.88	(0.65,1.20)	0.06	(0.04,0.11)	0.26	(0.15,0.45)
Pakistani	0.29	(0.25,0.33)	0.46	(0.39,0.54)	0.94	(0.82,1.08)	1.86	(1.56,2.21)	2.23	(1.97,2.52)	5.24	(4.44,6.19)	0.63	(0.55,0.73)	2.36	(1.96,2.84)
Bangladeshi	0.26	(0.16,0.43)	0.42	(0.26,0.68)	0.93	(0.62,1.39)	1.85	(1.22,2.81)	1.44	(1.01,2.05)	3.44	(2.37,5.00)	0.86	(0.58,1.26)	3.35	(2.21,5.06)
Other	0.15	(0.10,0.22)	0.21	(0.15,0.32)	0.43	(0.31,0.61)	0.73	(0.52,1.04)	0.27	(0.19,0.40)	0.51	(0.35,0.76)	0.24	(0.16,0.35)	0.57	(0.38,0.85)

**†**Model adjusted for woman’s age and marital status.

### Stratified LCA models for White British and Pakistani women only

For the White British women based on the model fit statistics and interpretability of the classes we chose the 4 class model. Likewise for the Pakistani women we also chose the 4 class model based on the model fit statistics and interpretability of the classes. We used the profiles of the different classes to describe the different groups and assign brief labels to these groups.

**Figure 2:**
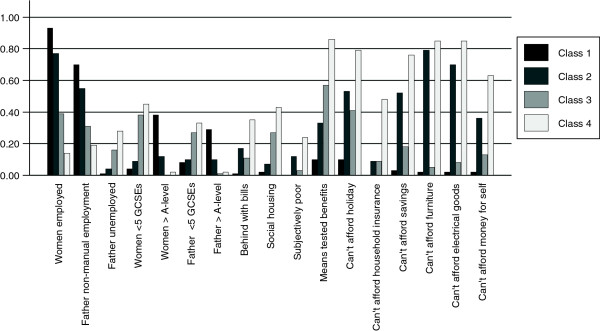
**Summary of selected predicted probabilities for levels of SEP indicators from 4 class model for White British women.** Class1: "Employed, educated and not materially deprived" (44%, n = 2038 (based on most likely class membership)). Class 2: "Employed, moderate education, materially deprived" (14%, n = 614). Class 3: "Low education, benefits not materially deprived" (23%, n = 992). Class 4: "Low education, benefits, subjectively poor and materially deprived" (18%, n = 836).

**Figure 3:**
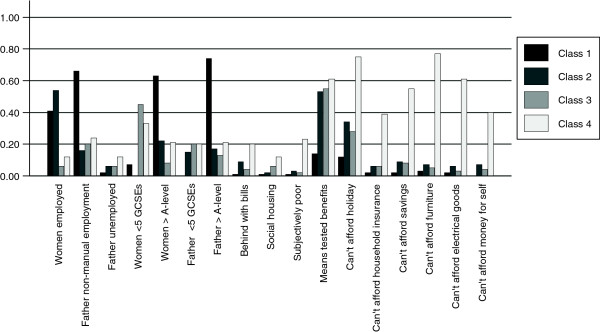
**Summary of selected predicted probabilities for levels of SEP indicators from 4 class model for Pakistani women.** Class1: "Educated, low benefits, not materially deprived" (22%, n = 1113 (based on most likely class membership)). Class 2: "Women employed, moderate education, benefits, not materially deprived" (17%, N = 935). Class 3: "Women not employed, low education, benefits, not materially deprived" (33%, n = 1642). Class 4: "Women not employed, moderate education, benefits, subjectively poor and materially deprived" (28%, n = 1427).

Tables 6 and 7 and Figures 2 and 3 describe the latent class profiles for the analysis stratified by ethnic group for White British and Pakistani women. Although for both ethnic groups a four class model was selected the characteristics and relative sizes of the four classes differed for each ethnic group. For the White British women the four classes can be described as (i) "Employed, educated and not materially deprived" (44%, n = 2038 (based on most likely class membership)), (ii) "Employed, moderate education, materially deprived" (14%, n = 614), (iii) "Low education, benefits not materially deprived" (23%, n = 992) and (iv) "Low education, benefits, subjectively poor and materially deprived" (18%, n = 836). For the Pakistani women the groups can be defined as (i) "Educated, low benefits, not materially deprived" (22%, n = 1113), (ii) "Women employed, moderate education, benefits, not materially deprived" (17%, N = 935), (iii) "Women not employed, low education, benefits, not materially deprived" (33%, n = 1642) and (iv) "Women not employed, moderate education, benefits, subjectively poor and materially deprived" (28%, n = 1427). For the two ethnic groups there were marked differences in the classes by the woman’s employment status and education. Within the White British group two classes can be described as materially deprived whereas within the Pakistani group only one class were materially deprived.

**Table 6:** **Description of latent classes from 4 class model solution for White British women**

Class	Size of class (n†)	Description
**"Employed, educated, not materially deprived"**	44% (n = 2038)	Women currently employed
		Father non-manual employment
		Women and fathers highly educated
		Up to date with bills
		Mortgage
		Not subjectively poor
		Not receiving means tested benefits
		Not materially deprived
**"Employed, moderate education, materially deprived"**	14% (n = 614)	Women currently employed
		Father manual and non-manual employment
		Women and fathers medium levels of education
		Moderate behind with bills
		Mortgage and private renting
		Moderate subjective poverty
		Moderate receipt of means tested benefits
		Material deprived - can’t afford holidays, money to replace good and savings
**"Low education, benefits not materially deprived"**	23% (n = 992)	Women moderate current employment
		Father manual and non-manual employment
		Women and fathers low levels of education, fathers education high don’t know response
		Moderate behind with bills
		Private renting and social housing
		Not subjectively poor
		High receipt of means tested benefits
		Not materially deprived
**"Low education, benefits, subjectively poor and materially deprived"**	18% (n = 836)	Women low current employment
		Father manual employment and unemployed
		Women and fathers low levels of education, fathers education high don’t know response
		Behind with bills
		Private renting and social housing
		Subjectively poor
		High receipt of means tested benefits
		Materially deprived

† n based on most likely class membership.

**Table 7:** **Description of latent classes from 4 class model solution for Pakistani women**

Class	Size of class (n†)	Description
**"Educated, low benefits, not materially deprived"**	22% (n = 1113)	Women moderate current employment
		Fathers non-manual employment
		Women and fathers highly educated
		Up to date with bills
		Mortgage and owns house outright
		Not subjectively poor
		Not receiving means tested benefits
		Not materially deprivation
**"Women employed, moderate education, benefits, not materially deprived"**	17% (n = 935)	Women current and previously employed
		Father manual employment and self-employed
		Women and fathers medium levels of education
		Moderate behind with bills
		Mortgage
		Not subjectively poor
		High receipt of means tested benefits
		Not materially deprived
**"Women not employed, low education, benefits, not materially deprived"**	33% (n = 1642)	Women low current employment
		Father manual employment and self-employed
		Women and fathers low levels of education, fathers education high don’t know response
		Up to date with bills
		Owns house outright
		Not subjectively poor
		High receipt of means tested benefits
		Not materially deprived
**"Women not employed, moderate education, benefits, subjectively poor and materially deprived"**	28% (n = 1427)	Women low current employment
		Father manual employment, high unemployment
		Women and fathers medium levels of education
		Behind with bills
		Social housing
		Subjectively poor
		High receipt of means tested benefits
		Materially deprived

† n based on most likely class membership.

## Discussion

Using data from a large multi-ethnic birth cohort we used latent class analysis to define an interpretable set of five classes with different SEP profiles. This method allows the combination of many different dimensions of SEP into one overall SEP measure. The five classes ranged in size from 16% to 29% of our cohort and included an affluent well educated class, a class of women who were mainly working and were not materially deprived, a group who were also working but were materially deprived, a group with high uptake of benefits but low levels of material deprivation and finally a group with low levels of employment and education, high uptake of benefits and high levels of material deprivation. Membership of these classes were associated with ethnicity and further analysis conducted separately for White British and Pakistani women found that different components of SEP aggregated differently in these two ethnic groups.

Other studies have also used LCA as a method to combine several dimensions of SEP into one indicator [10–14]. In this study the method provided a detailed description of the SEP profile of a multi-ethnic population of pregnant women with a level of detail that would not have been picked up using more traditional indicators of SEP, such as education or occupation. For example in the overall analysis there were two groups that were subjectively poor and did not have access to money to pay bills or buy and replace household goods. However, these groups had very different profiles in terms of employment and education suggesting that there are multiple pathways by which different determinants of SEP can lead to similarly poor outcomes.

The ethnic specific analysis further highlights that different measures of SEP are relevant in different ethnic groups. Although a four class model was selected for both the White British and Pakistani groups the characteristics of the four classes differed. The differences were in the woman’s employment status and education, housing, subjective poverty and material deprivation.

We used two different approaches to develop the SEP profiles for these women and the further use of these classes in epidemiological studies will depend upon the research question of interest. If investigating ethnic differences in health across ethnic groups then one overall SEP measure would be required, however if looking at differences in health within ethnic groups our results suggest that it may be more informative to develop ethnic specific SEP classes. Defining classes in other studies may be limited by the availability of the SEP measures in the study.

Our results are consistent with previous research showing that within South Asian ethnic groups there is heterogeneity in SEP with the Indian group found to have higher SEP than Pakistani and Bangladeshi groups [25]. Both the latter groups have been found to have the highest rates of poverty in the UK [26]. In our study we found that the Indian women were most likely to be in the most affluent group whilst Pakistani and Bangladeshi women were most likely to be in one of the more disadvantaged groups that had a high uptake of means tested benefits but were not materially deprived. This suggests that although they were disadvantaged on some aspects of SEP they are coping or had the support mechanisms available to support themselves financially. Other research has also found that factors relating to standard of living [27, 28] and asset based measures [5] are important measures of SEP in ethnic groups in addition to measures such as education and occupational social class.

### Strengths and limitations

To our knowledge this is the first time latent class analysis has been used to study the association between SEP and ethnicity in the UK. The key strengths are the large sample size and the inclusion of multiple measures of different dimensions of SEP. We used all available data on each category for each indicator including any missing responses ensuring the sample size was as large as possible. By including 19 different indicators of SEP including 11 questions about ownership of material deprivation items we were able to obtain a detailed description of how these women actually live and perceive deprivation in their day to day lives, this may not have been captured by using only traditional indicators of SEP.

There are some limitations to this work. Firstly we could not include a measure of income in our models for two reasons; over the recruitment period of the study different questions on income were asked, and the question asked about the income of the woman and her husband/partner not the household income. Many studies use equivalised household income however we cannot derive this from the information we have collected in this study.

We did not include a measure of occupational social class for the women. However there are problems with assigning social class to women as many women will not have an occupational social class if they are at home looking after the family. In our study we found that over a quarter of women had never been employed and so could not be assigned to an occupational social class. In our analysis we also did not capture any differences in household size and composition and this will vary by ethnic group. This could be further researched, along with exploring similarities and contrasts in what the measures of SEP mean to different ethnic groups.

The study offers a way of conceptualising SEP and ethnicity that reflects the complexity of the relationship between these two areas. Our findings support the hypothesis that before exploring the relationship between SEP, ethnicity and health adequate measures of SEP must be conceptualised and captured from real life data. The classes identified can now be linked to health outcomes over time to investigate the relationship between SEP, ethnicity and health. This method can also be used to capture changes in SEP profiles longitudinally.

## Conclusions

Latent class analysis is an approach that allows different aspects of an individual’s social and economic position to be considered in one multidimensional indicator and avoids narrow categorisation of SEP. These findings help us understand the role of different components of SEP within ethnic groups and the classes defined and described here can be used in future analyses investigating the relationship between ethnicity and health ensuring appropriate adjustment for SEP confounding.

## Abbreviations

SEP: Socioeconomic position
LCA: Latent class analysis
FIML: Full information maximum likelihood
RRR: Relative risk ratio.
